# Yolk sac-derived Pdcd11-positive cells modulate zebrafish microglia differentiation through the NF-κB-Tgfβ1 pathway

**DOI:** 10.1038/s41418-020-0591-3

**Published:** 2020-07-24

**Authors:** Ruimeng Yang, Ming Zhan, Miaomiao Guo, Hao Yuan, Yiqin Wang, Yiyue Zhang, Wenqing Zhang, Saijuan Chen, Hugues de The, Zhu Chen, Jun Zhou, Jun Zhu

**Affiliations:** 1 CNRS-LIA Hematology and Cancer, Sino-French Research Center for Life Sciences and Genomics, State Key Laboratory of Medical Genomics, Ruijin Hospital, Shanghai Jiao Tong University School of Medicine, 200025 Shanghai, PR China; 2 The Core Laboratory in Medical Center of Clinical Research, Department of Endocrinology, Shanghai Ninth People’s Hospital, State Key Laboratory of Medical Genomics, Shanghai Jiao Tong University School of Medicine, 200011 Shanghai, PR China; 3 Department of Urology, Shanghai Ninth People’s Hospital, Shanghai Jiao Tong University School of Medicine, 200011 Shanghai, PR China; 4 Division of Cell, Developmental and Integrative Biology, School of Medicine, South China University of Technology, 510006 Guangzhou, PR China; 5 Université de Paris 7/INSERM/CNRS UMR 944/7212, Equipe Labellisée No. 11 Ligue Nationale Contre le Cancer, Hôpital St. Louis, Paris, France; 6 Chaire d’Oncologie Cellulaire et Molecular, College de France, PSL Universite, Paris, France

**Keywords:** Cell biology, Molecular biology

## Abstract

Microglia are the primary immune cells in the central nervous system, which plays a vital role in neuron development and neurodegenerative diseases. Microglial precursors in peripheral hematopoietic tissues colonize the central nervous system during early embryogenesis. However, how intrinsic and extrinsic signals integrate to regulate microglia’s differentiation remains undefined. In this study, we identified the cerebral white matter hyperintensities susceptibility gene, programmed cell death protein 11 (*PDCD11*), as an essential factor regulating microglia differentiation. In zebrafish, *pdcd11* deficiency prevents the differentiation of the precursors to mature brain microglia. Although, the inflammatory featured macrophage brain colonization is augmented. At 22 h post fertilization, the Pdcd11-positive cells on the yolk sac are distinct from macrophages and neutrophils. Mechanistically, PDCD11 exerts its physiological role by differentially regulating the functions of nuclear factor-kappa B family members, P65 and c-Rel, suppressing P65-mediated expression of inflammatory cytokines, such as *tnfα*, and enhancing the c-Rel-dependent appearance of tgfβ1. The present study provides novel insights in understanding microglia differentiation during zebrafish development.

## Introduction

Cerebral white matter hyperintensities (WMH), also known as leukoaraiosis, are common in the aging population and are associated with increased risk of cognitive dysfunction, stroke, and neurodegenerative disorders including Alzheimer’s disease [1]. Perivascular inflammation is a prominent pathological feature in WMH, and WMH burden has been associated with circulating biomarkers of inflammation, including high-sensitivity C-reactive protein and Interleukin-6 [2–4]. Genome-Wide Association studies have helped identify many risk-associated single nucleotide polymorphisms (SNPs) related with WMH development and progression [5–7]. SNPs in *PDCD11* have showed significant associations with WMH burden, but their functional role remain undefined [5, 7].

Microglia, the innate immune cells of the central nervous system (CNS), enter the brain during early embryogenesis and play important roles in white matter pathology [8]. As influenced by their environment, microglia assume different molecular patterns ranging from M1 phenotype in secretion pro-inflammatory cytokines to M2 phenotype in inflammation resolution and tissue repair [9]. In the zebrafish embryo, macrophages develop early on the yolk sac (YS) expression *L-plastin* and spread in the cephalic mesenchyme at about 24 h post fertilization (hpf). After which they invaded brain and differentiated into microglia featured by lower expression of the *L-plastin*, and higher expression of the neurotrophic lipid carrier, apolipoprotein E at about 60 hpf [10, 11]. Factors like Colony-Stimulating Factor 1 Receptor (CSF1R) and neuronal apoptosis modulate the seeding of microglia precursors in the brain [12, 13]. But how intrinsic and extrinsic signals integrate in microglia differentiation remains largely unknown. Transforming growth factor-beta (TGF-β) family members are cytokine important for both innate and adaptive immune functions [14]. It is required for the maintenance of the microglia-specific homeostatic gene signature. Deletion of the TGF-β receptor in microglia resulted in rapid conversion of microglia toward an inflammatory macrophage phenotype [15, 16]. Although the importance of brain-derived TGF-β1 in shaping immune response in pathogenic conditions has been demonstrated, the origin of embryonic TGF-β1, especially for inducing microglia differentiation, remains unknown.

In this study, helped by zebrafish model organism, we demonstrated that *PDCD11*, a WHM susceptibility gene, helps promote embryonic microglia differentiation through Tgfβ1. Macrophages in *pdcd11* mutants showed increased expression of inflammatory genes and impaired upregulation of microglia signature genes. This imbalanced macrophage generation, likely caused by uncontrolled P65 activation, causes inflammatory gene upregulation and suppresses c-Rel-dependent Tgfβ1 expression under *pdcd11* deficiency.

## Results

### P53 independent inflammatory pathway activation in *pdcd11* mutants

**Fig. 1:**
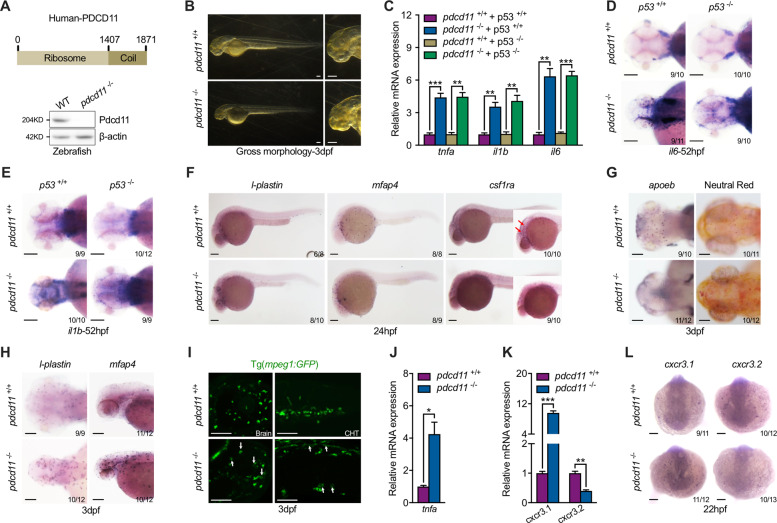
*Pdcd11* mutant zebrafish favor inflammatory macrophage generation. **a** Schematic diagram showing the two major function domains of PDCD11: N-terminal Ribosome domain (1–1407) and C-Terminal Coil domain (1408–1871). **b** Gross morphology of 3 dpf WT and *pdcd11* mutants. **c** qPCR confirmation of hyperactivated inflammatory pathway genes, including *tnfa*, *il1b*, and *il6*, in 22 hpf *pdcd11* mutants, which could not be restored by combined *p53* mutation. **d**–**e** *Il6* and *il1b* expression in the brain of WT or *pdcd11* mutants with *p53* mutated or not examined by WISH at 52 hpf. **f** WISH analysis of *l-plastin, mfap4*, and *csf1ra* expression in 24 hpf WT and *pdcd11* mutant embryos. Red arrows indicate *csf1ra-*positive cells in the brain. **g** Microglia development in 3 dpf WT and *pdcd11* mutants by WISH assessment of *apoeb* and Neutral Red staining. **h** *L-plastin* and *mfap4* expression pattern in 3 dpf WT and *pdcd11* mutants. **i** Morphology of macrophages in the brain and caudal hematopoietic tissue (CHT) were examined using the Tg(*mpeg1:GFP*) transgenic line. White arrows indicate the vacuolated macrophages found in the *pdcd11* mutant. **j** Increased *tnfa* mRNA expression in sorted macrophages Tg(*mpeg1:GFP*) from 60 hpf *pdcd11* mutants as compared with WT controls. **k** qPCR examination of the expression of macrophage-related genes *cxcr3.1* and *cxcr3.2* in 22 hpf WT and *pdcd11* mutants. **l** WISH analysis of *cxcr3.1* and *cxcr3.2* expression in 22 hpf WT and *pdcd11* mutants. The number positioned in the lower right corner of **d**–**h**, **l** represent the number of zebrafish embryos shown positive phenotypes versus the total number of embryos examined. Scale bar: 100 μm. Black horizontal lines indicate mean ± standard error of mean (SEM). Means ± SEM are shown for three independent experiments. ^*^P < 0.05; ^**^P < 0.01; ^***^P < 0.001 (Student’s *t* test).

Pdcd11 is highly conserved between species and mainly harbors two functional domains, including an N-terminal ribosome and Coil (Fig. 1a). To define the role of Pdcd11 in embryonic development, we generated a *pdcd11* mutant zebrafish line with a 7-bp deletion in the fourth exon by CRISPR-Cas9 nuclease (Fig. S1a). Antibody targeting the ribosome domain of Pdcd11 confirmed the complete loss of functional domains in *pdcd11* mutants (Fig. 1a). Perturbations of ribosome biogenesis often stabilize and activate genes related to the P53 apoptosis pathway [17–19]. Common with other reported ribosome defects, obvious developmental retardation and craniofacial malformation were observed in 3 dpf *pdcd11* mutants (Fig. 1b). Not surprisingly, P53 pathway genes were hyperactivated (Fig. S1b) and acridine orange (AO)-positive dead cells were also significantly increased in *pdcd11* deficient embryos from 22 hpf (Fig. S1c). To determine the contribution of the induction of apoptosis in the developmental defects of *pdcd11* mutants, *pdcd11* mutants in a *p53* knock-out background were also generated [20]. *P53* depletion favorably recovered AO cell numbers and P53 pathway genes in *pdcd11* mutants (Fig. S1d, e). However, cranial deformities, shorter body lengths, and survival rates were only partially restored (Fig. S1f, g), possibly suggesting that other P53 independent factors also contribute to the pathology of *pdcd11* mutants.

Interestingly, consistent with the perivascular inflammation a prominent pathological feature in WMH, we found that genes associated with inflammatory responses, like *il6*, *il1b*, and *tnfa*, were significantly increased, which otherwise failed to be restored by combined *p53* mutation (Fig. 1c). Using whole-mount in situ hybridization (WISH), the apparent increased expression of inflammatory genes in the brain of 52 hpf *pdcd11* mutants were detected, which also could not be recovered by the *p53* mutation (Fig. 1d, e).

### Macrophage generation imbalance in *pdcd11* mutants

Inflammatory gene expression was elevated in *pdcd11* mutants as early as 22 hpf. At this time, macrophages and neutrophils were the two major types of immune-related inflammatory cells identified in zebrafish. Macrophages initiate migration in the cephalic mesenchyme at about 24 hpf. Compared with that in WT embryos, *pdcd11* deficiency induced increased and earlier accumulation of macrophages in brain tissue as revealed by *mfap4* or pan-leukocyte marker *l-plastin* staining (Fig. 1f). The distribution pattern of *mpo*-positive leukocytes remained unaltered at this time (Fig. S2a). A previous study showed that apoptotic neuronal death drives the colonization of microglia precursors in the optic tectum [21]. Thus, we speculated that the advanced macrophage migration might be stimulated by increased neural apoptotic signaling. However, the combined *p53* mutation failed to restore the increased macrophage accumulation in the brains of *pdcd11* mutants, with accumulation actually being exacerbated (Fig. S2b). Interestingly, distinct from nonspecific macrophage markers, the expression of *csf1ra*, which was previously shown to be required for the migration and proliferation of microglia precursors [13, 22], was contrarily reduced in *pdcd11* mutants (Fig. 1f). Consistently, staining by either *apoeb* or Neutral Red revealed that mature microglia, which are tissue-resident macrophages in the brain, were completely absent in 3 dpf *pdcd11* mutants (Fig. 1g). At this time, macrophages stained for *mfap4* or *l-plastin* were abundant in the brain of *pdcd11* mutants, with an evidently enlarged and vacuolated morphology, which was also verified using a macrophage transgenic line Tg(*mpeg1:GFP*) (Fig. 1h, i). Macrophages acquire specific functional phenotypes that are manifest by secretion of inflammatory gene products and their morphological appearance. We found that the expression of *tnfa*, a well-established marker of M1 macrophages in zebrafish [23], was obviously increased in macrophages sorted from 60 hpf *pdcd11-*deficient embryos (Fig. 1j). In teleost fish, *cxcr3.1* and *cxcr3.2* macrophages show functional polarization towards M1 and M2, respectively [24]. We found that in 22 hpf *pdcd11* mutants, *cxcr3.1* expression was profoundly increased while that of *cxcr3.2* was reduced as compared with that in WT controls (Fig. 1k, l).

Similar to the failed restoration of inflammatory genes with *p53* mutation, simply inhibiting apoptosis could not restore the macrophage generation abnormalities observed in *pdcd11* mutant embryos (Fig. S2c, d). Moreover, no obvious aberrations in proliferation or apoptosis of myeloid progenitors were detected in *pdcd11* mutants at 22 hpf (Fig. S2e–h).

### Pdcd11-positive cells yield Tgfβ1 required for microglia differentiation

**Fig. 2:**
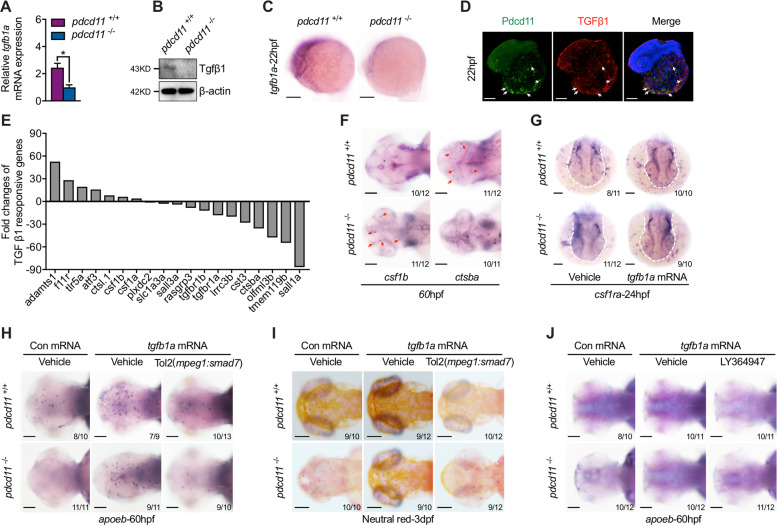
Reduced TGF-β1 signature genes in *pdcd11*-deficient macrophages. qPCR (**a**), western blot (**b**) and WISH (**c**) examination of the expression levels of *tgfb1a* or Tgfβ1 in 22 hpf zebrafish. **d** Immunofluorescence assessment of Pdcd11 and Tgfβ1 expression in 22 hpf zebrafish embryos. White arrows indicate the co-expressed signals found on the YS. **e** Fold changes of Tgfβ1-regulated genes expression in macrophages sorted from the brains of 60 hpf *pdcd11* mutants as compared with WT controls using Tg(*mpeg1:GFP*) transgenic line. **f**. Expression of *csf1b* and *ctsba* in 60 hpf WT and *pdcd11* mutants by WISH. Red arrows indicate the positive cells. **g** Effect of zebrafish *tgfb1a* mRNA overexpression on *csf1ra* expression in WT and *pdcd11* mutants. Effect of *tgfb1a* mRNA or combined *mpeg1* driven smad7 overexpression on microglia markers *apoeb* (**h**) or Neutral Red staining (**i**) in WT and *pdcd11* mutants. **j** The effect of LY364947 addition on *apoeb* expression in WT and *pdcd11* mutants with *tgfb1a* mRNA overexpression. The number positioned in the lower right corner of **f**–**j** represent the number of zebrafish embryos shown positive phenotypes versus the total number of embryos examined. Scale bar: 100 μm. Means ± SEM are shown for three independent experiments. ^*^P < 0.05 (Student’s *t* test).

Microglia differentiation requires step-wise-activated intrinsic transcription factors and circumstance-derived inducing factors, like Tgfβ1. Through comparing the RNA-seq data carried between 22 hpf WT and *pdcd11* mutants, we noticed that *tgfb1a* mRNA, a homolog of human *TGFB1*, was profoundly reduced in 22 hpf *pdcd11* mutant embryos (Table S1). The finding was verified by qPCR and western blot (Fig. 2a, b). Using WISH, we found that *tgfb1a* was highly expressed in cells on the YS at 22 hpf, at a location similar to Pdcd11, and that the expression of *tgfb1a* was obviously reduced with *pdcd11* deficiency (Fig. 2c). Otherwise the expression of *tgfb1b*, another orthologue of human *TGFB1* were lowly expressed at 22 hpf and no difference was detected with *pdcd11* depletion (Fig. S2i, j). Moreover, specially enriched Tgfβ1 in Pdcd11 expression cells was revealed using an immunofluorescence assay (Fig. 2d). Tgfβ1-induced signature genes in mouse microglia have been studied [25, 26]. Supporting the role of *pdcd11* in mediating Tgfβ1 activation in microglia, brain macrophages sorted from *pdcd11-*deficient Tg(*mpeg1:GFP*) line showed reduced expression of Tgfβ1-stimulated genes, including *sall1a*, *tgfbr1a* and *tgfbr1b*, and increased expression of Tgfβ1-suppressed genes like *tlr5a* and *ctsl.1* as compared with that in WT embryos (Fig. 2e). The elevation of *csf1b* and reduction of *ctsba* in microglia were also verified by WISH (Fig. 2f). Confirming the importance of *tgfb1a* reduction in contributing to microglia depletion in *pdcd11* mutants, *tgfb1a* overexpression restored both precursor and mature microglia numbers in *pdcd11* mutants (Fig. 2g–i). However, this rescue was compromised by the simultaneous repression of Tgfβ activation in macrophages by either LY364947, a TGFβR-I inhibitor addition, or overexpressing inhibitory cofactor smad7 under control of the *mpeg1* promoter, implying the importance of Pdcd11-derived Tgfβ in regulating macrophage development (Fig. 2h–j).

### Elevated P65 transcriptional activity in *pdcd11* mutants

**Fig. 3:**
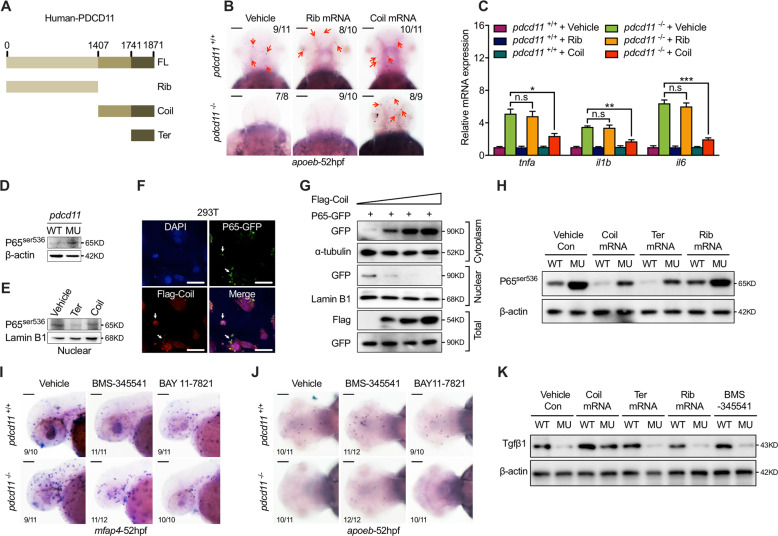
P65 hyperactivation in *pdcd11* mutants. **a** Schematic diagram showing different domains of PDCD11 consisting of Ribosome (Rib, 1-1407 aa), Coil (1408-1871 aa), and Terminal (Ter, 1742-1871 aa) domains. **b** Rescuing effects on *apoeb* positive microglia of *pdcd11* mutants with the Rib or Coil domain mRNA overexpression. Red arrows indicate the *apoeb* positive cells. Scale bar: 100 μm. **c** qPCR assessment of inflammatory genes expression in *pdcd11* mutants with Rib or Coil domain of PDCD11 overexpression. **d** Western blot examination of the 536-serine modified P65 expression in 22 hpf WT and *pdcd11* mutants. **e** Western blot examination of nuclear P65^536^ contents with the Coil or Ter overexpression in HEK293T cells. **f** Immunofluorescence assay showing the expression pattern of cells co-transfected with N-terminal Flag fused Coil and P65-GFP. White arrowheads indicate the colocalized Coil and P65 in the cytoplasm or nucleolus. Scale bar: 20 μm. **g** Representative image of three independent experiments showing the effect of increasing Coil transfection on nuclear/cytoplasm P65 content. **h** Representative image of three independent experiments showing P65^536^ levels in WT and *pdcd11* mutants with Coil domain (Coil), Terminal domain (Ter), or Ribosome domain (Rib) mRNA overexpression. **i** Appearance of *mfap4* expressing macrophages in *pdcd11* mutants treated with NF-κB inhibitor. Scale bar: 50 μm. **j** Rescuing effect of NF-κB inhibitors on *apoeb* positive microglia in *pdcd11* mutants. Scale bar: 100 μm. **k** Representative image of three independent experiments showing the rescue effects of Tgfβ1 levels with Coil domain (Coil), Terminal domain (Ter), Ribosome domain (Rib) mRNA or NF-κB inhibitor addition in 22 hpf *pdcd11* mutants. The number positioned in the upper right corner of **b** and lower right corner of **i**, **j** represent the number of zebrafish embryos shown positive phenotypes versus the total number of embryos examined. Means ± SEM are shown for three independent experiments. n.s not significant, ^*^P < 0.05; ^**^P < 0.01; ^***^P < 0.001 (Student’s *t* test).

Aside from the large N-terminal ribosome domain (Rib), Pdcd11 contains C-Terminal Coil domain (Coil), the function of which has not been defined (Fig. 3a). To investigate the molecular mechanism, we first overexpressed Rib and Coil. While Rib overexpression faithfully restored P53 pathway genes, the disturbances of macrophage generation and inflammation pathway activation could only be reversed with Coil overexpression in *pdcd11* mutants (Fig. 3b, c and S3a–c). A short C-Terminal fragment (Ter) reportedly could interact with P65 (Fig. 3a), one member of the NF-κB family [27, 28]. The NF-κB signaling pathway participates in modulating tissue immune homeostasis. Under normal circumstances, P65 is retained in the cytoplasm by the inhibitory protein IkappB and is available to the proteasome degradation pathway, which avoids uncontrolled inflammation activation. Upon stimulation, P65 is post-translationally modified by phosphorylation at the 536-serine residue, which obviates the interaction with IκB. P65 can then translocate into the nucleus to activate the downstream signaling pathway. We found that 536-serine phosphorylated P65 was obviously increased in *pdcd11* mutants (Fig. 3d). In vitro overexpression of Coil or simply the terminal fragment Ter efficiently reduced nuclear 536-serine phosphorylated P65 (Fig. 3e). Interestingly, we found that in vitro transfected P65 and Coil mostly colocalized in the cytoplasm or in the nucleolus, which correlated with reduced P65 transcriptional activity [29, 30] (Fig. 3f). The cytoplasm and nuclear P65 content with Coil overexpression were then examined respectively by western blot. Increasing Coil transfection obviously increased the P65 cytoplasm content, while correspondingly reducing the nuclear P65 level (Fig. 3g). Moreover, increased 536-serine phosphorylated P65 in the *pdcd11* mutants were restored by either Coil or Ter domain overexpression, but not by Rib domain overexpression (Fig. 3h). To determine whether the rescuing effects of Coil relied on its capacity in suppressing P65 activation, two NF-κB inhibitors, BMS-345541 and BAY 11-7821, were used [31]. Treatment with either BMS-345541 or BAY 11-7821 reduced 536-serine phosphorylated P65, and inflammatory genes and macrophages also returned to normal in *pdcd11* mutants (Fig. 3i and S3d, e). A correctly regulated inflammasome cascade is required for the maintenance of microglia homeostasis [32]. However, microglia number or the TGFβ1 level could not be rescued by BMS-345541 or BAY 11-7821 treatment (Fig. 3j, k), suggesting that other P65 independent mechanisms might be utilized by Coil in regulating microglia generation.

Interestingly, while Coil overexpression restored both macrophage appearance and inflammatory genes in the *pdcd11* mutants, similar efficacy was not detected with either Coil overexpression in pan neuron cells under *Huc* promoter (also named embryonic lethal, abnormal vision-like, neuron-specific RNA-binding protein 3, *elavl3*) [33] or macrophages under control of the *mpeg1* promoter [34] (Fig. S3c, f).

### Pdcd11 enhances c-Rel/p105 nuclear transport

**Fig. 4:**
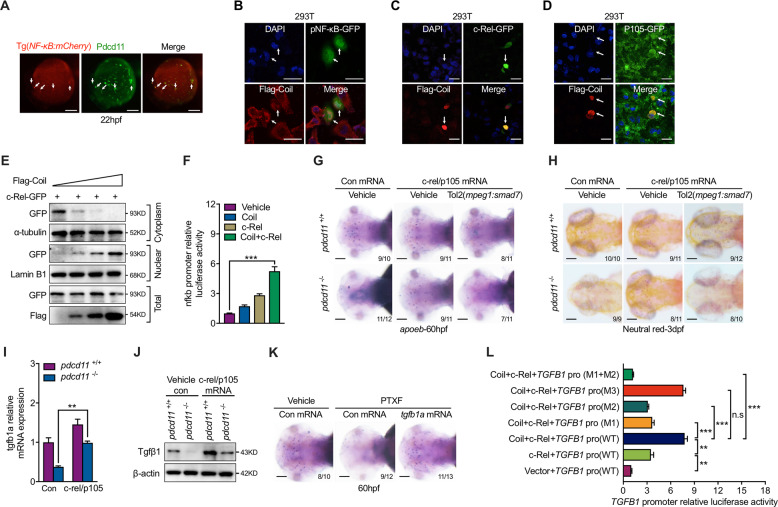
PDCD11 promotes c-Rel mediated NF-κB activation. **a** IF assay of Pdcd11 expression in Tg(*NF-κB:mCherry*) transgenic line. White arrowheads indicate the colocalized cells on the YS at 22 hpf. Scale bar: 100 μm. **b** In vitro transfection of plasmid containing NF-κB recognition motif driven GFP and Flag-Coil. White arrows indicate that cells with nuclear Coil expression displayed NF-κB activation. Scale bar: 20 μm. Representative image show cells co-transfected with Flag-Coil and c-Rel (**c**) or P105 (**d**). White arrows indicate that cells with nuclear expressed Coil and c-Rel (**c**) or P105 (**d**). Scale bar: 20 μm. **e**. Western blot showing the effect of increasing Coil transfection on nuclear/cytoplasm c-Rel content. **f** Influence of Coil and c-Rel transfection on luciferase activities driven by the NF-κB-binding site. Microglia numbers in WT and *pdcd11* mutants examined by *apoeb* (**g**) or Neutral Red staining (**h**) with *c-rel* and *p105* mRNA or combined *mpeg1* promoter driven *smad7* plasmid injection. Scale bar: 100 μm. qPCR (**i**) and western blot (**j**) assessment of *tgfb1a* or Tgfβ1 level in 22 hpf WT and *pdcd11* mutants with *c-Rel* and *p105* mRNA overexpression. **k** Microglia numbers in embryos with c-Rel inhibition (PTXF) or combined *tgfb1a* mRNA complementation. Scale bar: 100 μm. **l** Reporter assay showing the regulation of *TGFB1* promoter by Coil and c-Rel transfection. *TGFB1* WT promoter containing three c-Rel-binding sites (5′-ccGGGGcacccccc-3′, −761 bp to TSS; 5′-ggGGGGacgccccgt-3′, −776 bp to TSS; 5′-aaGGGAcccccctcg-3′, −1019 bp to TSS). The three binding motifs were respectively mutated and designated as M1 (5′-ccAAAAcacccccc-3′, −761 bp to TSS), M2 (5′-ggAAAAacgccccgt-3′, −776 bp to TSS), M3 (5′-aaAAACcccccctcg-3′, −1019 bp to TSS). The number positioned in the lower right corner of **g**, **h**, **k** represents the number of zebrafish embryos shown positive phenotypes versus the total number of embryos examined. Means ± SEM are shown for three independent experiments. n.s not significant, ^*^P < 0.05; ^**^P < 0.01; ^***^P < 0.001 (Student’s *t* test).

Transgenic zebrafish with NF-κB recognition sequences driven fluorescent protein was utilized to study NF-κB activity in zebrafish [35]. Helped by this transgenic line, we were surprised to find that although PDCD11 suppressed P65 activation, NF-κB was otherwise activated in Pdcd11-expressing cells on the YS at 22 hpf (Fig. 4a). In comparison with cytoplasm Coil expressed cells, cells with nuclear Coil expression were prone to activate EGFP under control of the NF-κB recognition sequences (Fig. 4b and S4a). Among the five members of NF-κB family, P65, c-Rel, and Rel-B harbor the transcription activation domain (TAD), which binds NF-κB recognition sequences and activates the expression of downstream genes [36, 37]. The findings prompted us to speculate that this activation of the NF-κB-binding element relied on the transcriptional activity of other members of the NF-κB family. Indeed, different from P65, Coil colocalized with c-Rel and P105 in the nucleus and Coil overexpression promoted the nuclear retention of c-Rel (Fig. 4c–e and S4b, c). A luciferase assay showed that Coil co-expression enhanced the capacity of c-Rel in activating the NF-κB-binding element (Fig. 4f). Confirming the importance of reduced nuclear c-Rel/P105 in leading the microglia deficiency in *pdcd11* mutants, we found that c-Rel and p105 co-overexpression recovered microglia numbers as manifested by either *apoeb* or Neutral Red staining (Fig. 4g, h). These rescue effects were largely compromised when repressive smad7 was co-expressed in macrophages, suggesting that c-Rel/P105 functions upstream of TGFβ signaling (Fig. 4g, h). Consistently, the expression level of Tgfβ1 was also increased with c-Rel and p105 mRNA overexpression (Fig. 4i, j). Injection of the xanthine derivative pentoxifylline (PTXF), which selectively degrades c-Rel [38], also reduced the number of microglia in WT embryos. The reduction could also be recovered by the injection of *tgfb1a* mRNA (Fig. 4k). In a previous study, chromatin immunoprecipitation combined with microarray technology (ChIP/Chip) examination of mitogen-stimulated T cells Rel/NF-kappa-B revealed potential Rel/NF-κB recognition motifs in the promoter region of *TGFB1* [39]. To further determine the regulatory role on *TGFB1* by Coil and c-Rel, HEK293 cells were examined using a luciferase reporter assay. Coil co-expression enhanced the capacity of c-Rel in stimulating the *TGFB1* promoter. This enhancement was abolished by the combined M1 and M2 c-Rel-binding site mutation (Fig. 4l). These results suggest that Coil and c-Rel promotes *tgfb1 e*xpression in early zebrafish embryos.

**Fig. 5:**
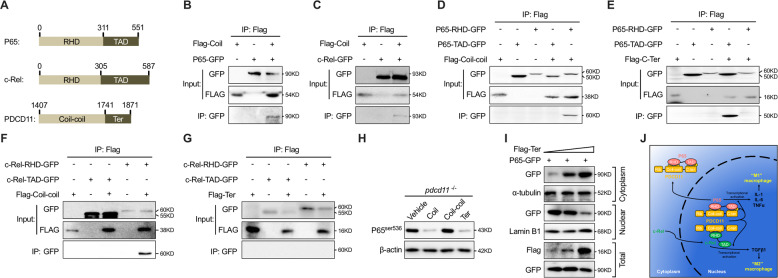
Different interaction pattern between P65 or c-Rel and PDCD11. **a** Schematic diagram showing the domain structures of human P65, c-Rel, and PDCD11-Coil. **b** Immunoprecipitation (IP) assay of the interaction between P65-GFP and Flag-Coil. **c** IP assay of the interaction between c-Rel-GFP and Flag-Coil. **d** IP assay of the interaction between the Coil–coil fragment of PDCD11 with the RHD domain or TAD domain of P65. **e** IP assay of the interaction between the terminal (Ter) fragment of PDCD11 with the RHD domain or TAD domain of P65. **f** IP assay of the interaction between the Coil–coil fragment of PDCD11 with the RHD domain or TAD domain of c-Rel. **g** IP assay of the interaction between the terminal (Ter) fragment of PDCD11 with the RHD domain or TAD domain of c-Rel. **h** Western blot examination of the influential role of overexpression Coil, Coil–coil and Terminal of PDCD11 on P65^ser536^ expression in 22 hpf *pdcd11* mutants. **i** Western bot show the effect of increasing Ter transfection on nuclear/cytoplasm P65 content in 293T cells. **j** Schematic diagram showing the working model for PDCD11-mediated regulation of c-Rel and P65. Under normal circumstances, on one hand, the Ter fragment of PDCD11 (yellow) binds and masks the phosphorylation site of P65 (pink), which is required for its nuclear translocation and transcriptional activation; on the other hand, the only existing interaction between the coil–coil of PDCD11 and the RHD domain of c-Rel contrarily promotes c-Rel (green) nuclear retention for activating TGFβ1 expression, which drives macrophage differentiation toward microglia with “M2” properties. However, cells deficient in *PDCD11* show increased P65 nuclear translocation, which tends to lead to activation of inflammation-related genes, such as *IL-1*, *IL-6*, and *TNFα*. These cytokines then facilitate “M1” macrophage differentiation.

Since P65 and c-Rel are structurally similar and both harbor an N-terminal Rel homology domain (RHD) and C-Terminal TAD domain, it was of interest to investigate how PDCD11 distinguished the two (Fig. 5a). Co-IP examination revealed that both P65 and c-Rel could interact with the Coil domain of PDCD11 (Fig. 5b, c). The Coil domain was further divided into the Coil–coil and Ter fragment, which displayed a different interaction pattern with P65 and c-Rel. Since the RHD domain of P65 and c-Rel interact with the Coil–coil region of PDCD11, the Ter fragment failed to bind to the TAD domain of c-Rel as observed with P65 (Fig. 5d–g). This result was reasonable since, because the RHD domains of NF-κB family members are similar, their TAD domains are much more distinct. The interaction of Ter with the TAD domain of P65 might cover the phosphorylation site required for P65 transcriptional activation. The mere overexpression the Ter fragment could suppress the P65 activation in *pdcd11* mutants, otherwise the Coil–coil fragment overexpression failed to do so (Fig. 5h). Confirming the importance of Ter fragment in inhibiting P65 activation, the overexpression of this fragment also inhibited P65 nuclear translocation (Fig. 5i). Thus, the differential interactions between the TAD domains and PDCD11 might contribute to the different regulation mode of P65 and c-Rel by PDCD11 (Fig. 5j).

### YS Pdcd11-expressing cells are derived from Pu.1-progenitors

**Fig. 6:**
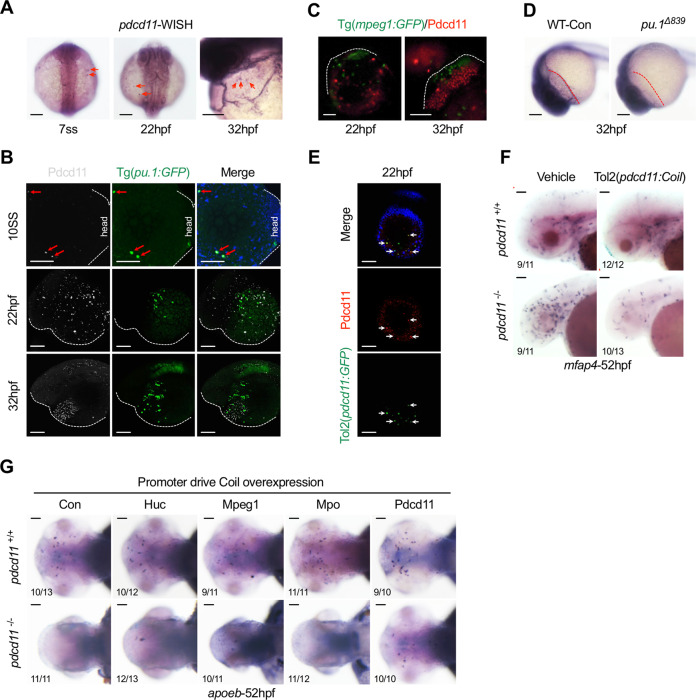
Pdcd11 expression pattern examined during zebrafish embryonic development. **a** WISH assessment of *pdcd11* expression in zebrafish at 7-ss, 22 hpf, and 32;hpf. The red arrows indicate *pdcd11* signals on the YS and pericardial cavity. **b** Immunofluorescence staining of Pdcd11 in Tg(*pu.1:GFP*) zebrafish. The red arrows indicate colocalized Pdcd11 and Pu.1 expressing cells on the YS at 10-ss. **c** Pdcd11 expression in 22 hpf and 32 hpf Tg(*mpeg1:GFP*) zebrafish assayed by immunofluorescence staining. **d** WISH examination of *pdcd11* expression in *pu.1* deficient embryos. The red dotted lines denote the pericardial cavity underneath the brain where *pdcd11* expressed. **e** IF assessment of the co-expression between GFP driven by *pdcd11* promoter and endogenous Pdcd11. White arrowheads indicate the colocalization signal on the 22 hpf YS. **f** The expression pattern of *mfap4-*positive macrophages in the 52 hpf brain with *pdcd11* promoter driven Coil overexpression. **g** Rescue effects of *apoeb* positive microglia with Coil overexpressed under different promoters including *Huc*, *mpeg1*, *mpo*, and *pdcd11*. The number positioned in the lower left corner of Fig. 6f, g represent the number of zebrafish embryos shown positive phenotypes versus the total number of embryos examined. Scale bar: 100 μm.

By WISH, we found that aside from the obvious expression signal in the neuron epithelium, scattered cells on the YS were also detected from 7-somite stages (ss) (Fig. 6a). At 32 hpf, *pdcd11*-positive cells had accumulated in the pericardial cavity, the site of inflow of all blood cells (Fig. 6a). In zebrafish, myeloid progenitor cells expressing Pu.1 emerged within the anterior lateral plate mesoderm (ALPM) at the 5-ss and then migrated into the rostral blood island (RBI) and onto the YS. This was followed by differentiation of the cells into macrophages and neutrophils, which then seeded the brain from approximately 24 hpf. Through use of the zebrafish Tg(*Pu.1:GFP*) transgenic line, we found that at 10-ss in the ALPM region, Pdcd11 was activated in a subset of Pu.1-GFP-positive cells (Fig. 6b). However, this co-expression pattern reduced at 22 hpf and thereafter remained absent, suggesting later silencing Pu.1 expression (Fig. 6b). The expression of Pdcd11 in macrophages and neutrophils was also examined using Tg(*mpeg1:GFP*) and Tg(*mpo:GFP*) transgenic lines, respectively. Mpeg1-positive macrophages appeared to be closely surrounded by Pdcd11-positive cells in the pericardial cavity underneath the brain at 32 hpf, but no colocalizations were detected (Fig. 6c). Similarly, no colocalizations between Pdcd11 and mpo-positive neutrophils were detected (Fig. S4d). Intriguingly, in *pu.1-*deficient embryos, while *pdcd11* expression in the neuron epithelium remained unchanged, its expression in the pericardial cavity was disappeared, implying that the scattered *pdcd11*-expressing cells observed on the YS were Pu.1-dependent (Fig. 6d). To verify the *pdcd11* expression pattern, the 1.86 kb genomic region upstream of the translation initiation site of zebrafish *pdcd11* was cloned into a transposon vector containing EGFP. The *pdcd11* promoter-driven GFP-expressing cells on the YS strongly colocalized with endogenous Pdcd11 at 24 hpf in an immunofluorescence assay (Fig. 6e). Overexpression of Coil under the control of the 1.86 kb *pdcd11* promoter reversed the macrophage imbalance in *pdcd11* mutants, with the number of inflammatory macrophages reduced and that of microglia increased (Fig. 6f, g). This rescue effect was not observed in embryos overexpressing Coil overexpression under the control of neuron (*Huc*), macrophage (*Mpeg1*), or neutrophil (*Mpo*) specific promoters (Fig. 6g). Collectively, these results suggest that scattered Pdcd11-expressing cells may be derivatives of Pu.1-positive progenitors, but that they are distinct from previously identified neutrophils or macrophages.

## Discussion

WMH examined by magnetic resonance imaging have been used to describe WM changes related with cognitive dysfunction, stroke injury, cerebral small vessel disease, and neurodegenerative disorders [40]. Key cellular components of the gliovascular unit including astrocytes, oligodendrocytes, pericytes and microglia. It was suggested that microglia within the WM be associated functions like surveillance at the blood–brain barrier and clearance debris [41, 42]. In this study, helped by zebrafish model organism, we found that deficiency of *pdcd11*, a WMH susceptibility gene, led to completely lost of mature microglia in the brain. In mouse, helped by in vivo lineage tracing, it was found that adult microglia derive from primitive myeloid progenitors that arise before embryonic day 8 [43]. However, as with zebrafish, multiple waves of microglia exist and originate from distinct hematopoietic precursors were identified [44]. Despite the different ontogeny of primitive and adult microglia between mouse and zebrafish identified, the regulators underlying the differentiation of EMP progenitors toward microglia remain undefined. TGF-β family members are cytokine required for the maintenance of the microglia-specific homeostatic gene signature. Deletion of the TGF-β receptor in microglia resulted in rapid conversion of microglia toward an inflammatory macrophage phenotype [15, 16]. Mice harboring mutated *TGFB1* exhibit an acute wasting syndrome followed by death [45]. The most prominent lesions are tissue necrosis in specific organs and multifocal, mixed inflammatory cell infiltration into numerous organs [46]. Despite accumulating evidence for local expression of TGF-β1 in the nervous system during inflammation, the cellular source of TGF-β1, which drives microglia differentiation is unknown. *Pdcd11*-deficient embryos exhibit brain necrosis and organ inflammatory cell infiltration, which is very similar to what is observed with *TGFB1*-mutated mice. Moreover, Pdcd11-expressing cells on the YS colocalized with TGF-β1-positive cells at 22 hpf on the YS. Both the mRNA and protein levels of TGF-β1 were significantly reduced with *pdcd11* depletion. Importantly, overexpression of TGF-β1 under the control of the *pdcd11* promoter faithfully recovered the microglia numbers in *pdcd11* mutants. Thus, our observations support the notion that during the early development in zebrafish, Pdcd11-expressing cells might be one of the major source of TGF-β1 to induce microglia maturation.

In studying the property of YS Pdcd11-expressing cells, we found that these cells are distinct from initially identified macrophages and neutrophils, but they are also derived from Pu.1-expressing progenitors and require Pu.1. The NF-κB family member possesses distinct biological functions and controls the expression of different target genes, manifesting as unique developmental phenotypes between NF-κB/Rel family member knock-out mice. The distinct and opposing functions of P65 and c-Rel in regulating cellular functions, such as proliferation and apoptosis, have been revealed in several different models [47, 48]. In P105 and c-Rel double knock-out mice, while peripheral T-cell populations developed normally, follicular, marginal zone, and CD5^+^ peritoneal B-cell populations were all reduced, suggesting the particular requirement of P105 and c-Rel in these cell types [49]. It was reported that B1a cells are abundant in the neonatal mouse brain and promote oligodendrogenesis by regulating the proliferation of oligodendrocyte-precursor cells and the number of microglia [50]. As the definitive markers for B1a lymphocytes in zebrafish are elusive, other model organism like mice might be useful for our understanding Pdcd11-positive cells in the future. A recent report in studying the *mpeg1.1:GFP* reporter line, it found that beside macrophages, a subpopulation of B-lymphocytes is marked by *mpeg1.1* reporters in most adult zebrafish organs, with highly expressed *cd79a*, *ighm*, and *ighd* [51]. This would also be helpful for our future examination of this transgenic line in study the property of Pdcd11-positive cells.

## Materials and methods

### Experimental model

Zebrafish maintenance and staging were performed as previously described [52]. The zebrafish facility and study were approved by the Institutional Review Board of the Institute of Health Sciences, Shanghai Institutes of Biological Sciences, Chinese Academy of Sciences (Shanghai, China), and the methods were carried out in accordance with the approved guidelines. The *Tg(pu.1: GFP)* [53], *Tg(mpeg1: GFP)* [54], *Tg(mpo: GFP)* [55], *P53*^zdf1/zdf1^ mutants [20, 56], *pu.1*^*Δ839*^ zebrafish lines were used.

### *Pdcd11* knock-out zebrafish line generation

For CRISPR-Cas9 mediated *pdcd11* knock-out zebrafish generation, guide RNA (gRNA) targeting exon4 of *pdcd11* (5′-GGTCTCCCGAGCGGCCTCGT-3′) was designed using the online tool, ZiFiT Targeter software (http://zifit.partners.org/ZiFiT), and was synthesized by cloning the annealed oligonucleotides into the sgRNA expression vector as previously described [57]. The mutant line is screened by Bsrbi enzyme (NEB) cutting and confirmed by Sanger sequencing.

### mRNA synthesis, and microinjection

mMACHINE Kit (Ambion) was used for mRNA transcription. For promoter-driven Coil or smad7 expression in zebrafish, 30 ng/μl transposase mRNA was coinjected.

### Chemical treatment

PTXF (Abcam) was injected (0.5 ng) into one-cell stage embryos. BMS-345541 (Selleckchem, 10 μM), BAY 11-7821 (Selleckchem,10 μM), and LY364947 (Selleckchem, 10 μM) were used for treating embryos from 18 hpf.

### Plasmids

The Tol2(*NFκB:EGFP*) plasmid was obtained from Addgene [35]. The 1.86-kb sequence immediately proximal to the *pdcd11* 5′ untranslated region was amplified and cloned into a modified pT2cfosGW vector containing the zebrafish Tol2 transposable element [35]. The resulting construct was injected, together with transposase mRNA, into one-cell stage WT TU embryos [58]. The full length PDCD11 was cloned into vector pcDNA3.1. The C-coil, C-coil–coil, and C-Ter domain of human PDCD11 were amplified and cloned into vector pCS2^+^. Full-length human c-Rel, P65, and P105 were cloned into pCS2^+^ with GFP fused in the C-terminus. The RHD and TAD domain of c-Rel and P65 were amplified from the full-length vector and cloned into pCS2^+^. Full-length zebrafish *tgfb1a* was amplified from zebrafish cDNA and cloned into the pCS2^+^ vector. *Mpeg1* promoter driven C-coil and smad7 were cloned by replacing dendra in vector Tol2(*mpeg1:dendra2*) (Addgene, 51462). Tol2(*mpx:Coil*) was cloned by replacing the Lifeact-Ruby (Cla1 and Sal1) with Coil in Tol2(*mpx:Lifeact-Ruby*) (Addgene, 45246). Tol2(*huc:Coil*) was also obtained by gateway cloning using p5E-Huc (Addgene, 72640).

### WISH

WISH was performed as previously described [59]. Probes for *l-plastin*, *csf1ra,* and *mpo* were used as previously described [10, 60, 61]. Antisense probes, including *il6*, *il1b*, *mfap4*, *apoeb*, *pdcd11*, *csf1a*, *ctsba*, *tgfb1a*, *tgfb1b, cxcr3.1*, and *cxcr3.2* were transcribed in vitro from PCR products amplified from zebrafish cDNA with the T7 transcriptase binding sequence added to the reverse primers. Primers used are shown in Table S2.

### Neutral Red, AO, TUNEL, pH3, and immunofluorescence assay

For Neutral Red staining, zebrafish larvae collected at 60 hpf and 3 dpf were soaked in Neutral Red (Sigma-Aldrich, N6264; 2.5 mg/mL) overnight at 28.5 °C. For AO staining, embryos recovered at 22 hpf were incubated in 10 μg/mL AO stain (Sigma-Aldrich) dissolved in E3 medium in the dark for ~30 min. TUNEL staining was performed as previously described [62]. pH3 and immunofluorescence staining were carried out using the following antibodies: PDCD11 (Sigma-Aldrich; HPA017924; 1:100); pH3 (Santa Cruz Biotechnology; 1:500), TGF-β1 (Sigma-Aldrich, T0438; 25 μg/mL)

### qPCR analysis

After treatment with DNAaseI (Ambion), RNA was prepared using Trizol reagent (Invitrogen, H10522) and then subjected to cDNA synthesis using a cDNA synthesis kit (ABI). Real-time PCR was performed using a Fast Start Universal SYBR^®^ Green Master Rox probe (Roche Applied Science, 13800300) and Mastercycler thermal cycler (Eppendorf, 22331). Primers are summarized in Table S2.

### Western blot, nuclear extraction, and co-IP

Zebrafish embryos at the indicated developmental stages were de-yolked and homogenized in lysis buffer mixed with proteinase inhibitor as previously described [63]. Nuclear proteins were extracted by the NE-PER^™^ Nuclear and Cytoplasmic Extraction Reagents (Thermo Fisher Scientific, 78833). For the co-IP assay, proteins were purified in RIPA buffer (50 mM Tris, 150 mM NaCl, 10% glycerol, 5 mM MgCl_2_, 0.5% NP40, and Roche cocktail protease inhibitor) 48 h after transfection, and mixed with anti-c-Flag agarose affinity gel antibody (Sigma-Aldrich, A220). After incubation at 4 °C overnight, beads were collected for western blotting. Samples were subjected to sodium dodecyl sulfate–polyacrylamide gel electrophoresis and immunoblotting according to standard protocols. The antibodies used were anti-GFP (Sigma-Aldrich, G1544), anti-Flag (Sigma-Aldrich, F7425), anti-PDCD11 (Sigma-Aldrich; HPA017924), anti-P65^ser536^ (Cell Signaling Technology, 3031), anti-α-Tubulin (Sigma-Aldrich, T9026), anti-β-Actin (Cell Signaling Technology, 8457), and anti-LaminB1 (Cell Signaling Technology, 13435).

### Cell culture and dual-luciferase reporter assay

Cells were cultured in a humidified incubator at 37 °C in the presence of 5% CO_2_. 293T and Hela cells obtained from ATCC were cultured in DMEM medium supplemented with 10% fetal bovine serum, 100 IU/mL penicillin, and 100 μg/mL streptomycin (Gibco). Identity of cell lines were validated by STR analysis. Mycoplasma testing was routinely performed on all cells used in the study, and confirmed to test negative. For cell transfection, Effectene transfection reagent (QlAGEN, 1012829) was used according to the manufacturer’s instructions. NF-κΒ binding motif driven luciferase reporter and human *TGFB1* (1.3-kb upstream the transcription start site) was used. C-Coil (150 ng) and/or c-Rel (150 ng) as well as Renilla constructs (5 ng) were transfected into 293 T cells (60%–70% confluence per well in 24-well plates) and luciferase activities were assessed according to the manufacturer’s instructions (Promega). Site mutagenesis was carried on the *TGFB1* promoter (Invitrogen, A13282)

### Cell sorting and cytology

Tg (*mpeg1:EGFP*) transgenic embryos at 60 hpf were connected and digested with 0.5% trypsin (Gibco) for 15 min at 37 °C to analyze microglia gene signature. Sixty hours post fertilization zebrafish brain was cut before digestion. After centrifugation at 400 × *g* for 5 min, two washes with PBS, and passage through a 40 μm nylon mesh filter, the single cell suspension was subjected to fluorescence-activated cell sorting using a MoFlo device (DakoCytomation). EGFP positive cells were used for downstream qPCR assay.

### Cell IF assay

Cell IF were carried as previously described. Cells grown to 60% confluence on coverslips were first fixed and blocked and then incubated with mouse anti-FLAG (Sigma-Aldrich, F7425). Secondary antibody goat anti-mouse, Alexa Fluor 594 (Invitrogen).

### Imaging

Zebrafish IF stain images were taken using an Olympus FV1000 scanning confocal microscope. The confocal images were captured with an UPLSAPO 40× or 60× objective. Immuno-stained cell images were collected using a Leika with an UPLSAPO 60× objective.

### RNA-sequencing (RNA-seq)

Total RNA from 22 hpf WT and *pdcd11* mutants was collected respectively and subjected to RNA-seq. Shanghai Novel Bioinformatics Company provided the deep-sequencing service.

### Statistical analysis

For all experiments carried, zebrafish embryos were randomized distributed to each group. The outcome of experiments were assessed according to phenotypic changes. Statistical analyses were conducted using the Student’s *t* test (two-sided) or log-rank tests. Statistical significance was taken to be *P* < 0.05.

## Supplementary information

Supplementary Figure legends

Supplemental Figure 1

Supplemental Figure 2

Supplemental Figure 3

Supplemental Figure 4

Supplemental Table 1

Supplemental Table 2
