# Supplementary material for: Yolk sac-derived Pdcd11-positive cells modulate zebrafish microglia differentiation through the NF-κB-Tgfβ1 pathway
Source: Cell Death Differ. 2020 Jul 24;28(1):170–83. doi: 10.1038/s41418-020-0591-3 (PMC7853042; doi:10.1038/s41418-020-0591-3)
Supplement: Supplementary file 1 — Supplementary Figure legends [file 41418_2020_591_MOESM1_ESM.docx]

**Supplementary Figure legends**

**Figure S1. Phenotype examinations of *pdcd11* and *p53* double mutant embryos.**

A. The upper portion is a schematic diagram showing the target of gRNA in the exon4 of zebrafish *pdcd11*. The lower portion is the gel electrophoresis verification of the mutation by *Bsrb*I cutting.

B. P53 pathway genes including *mdm2*, *p21*, and *baxa* in 22hpf WT and *pdcd11* mutants examined using qPCR.

C. Acridine orange (AO) staining of 24hpf WT and *pdcd11* mutants. On the right are the magnified images showing increased AO positive cells in the brain of *pdcd11* mutants.

D. AO staining showing apoptotic cells in 24hpf zebrafish brain with corresponding *pdcd11* and/or *p53* mutations.

E. qPCR examination of the expression of apoptotic genes in 24hpf *pdcd11* and/or *p53* mutants.

F. Gross morphology analysis of 3dpf *pdcd11* and/or *p53* double mutants. Cranial deformities are indicated by white arrowheads.

G. Kaplan-Meier survival curves showing reduced survival rate of *pdcd11* mutants as compared with sibling WT control, which could only be partially restored with combined *p53* mutation. 20 embryos were included for each group.

Scale bar: 100μm. Means ± SEM are shown for three independent experiments. n.s, not significant, ^*^*P* < 0.05; ^**^*P* < 0.01; ^***^*P* < 0.001 (Student’s t-test).

**Figure S2. Rescue effects of *P53* mutation on abnormal macrophage behavior in *pdcd11* mutants.**

A. WISH analysis of the expression of *mpo* labeled neutrophils in WT and *pdcd11* mutants.

B. WISH analysis of *mfap4* positive macrophages in the brain of 24hpf embryos with combined *pdcd11* and *p53* mutation. White arrowheads indicate the *mfap4* positive cells in the brain.

C-D. Rescue effects of *p53* mutation on 52hpf *mfap4* expressing macrophages (C) and 60hpf *apoeb* positive microglia (D) in *pdcd11* mutants.

E-F. The numbers of pH3 and Pu.1:GFP double positive cells (n = 20 each) calculated in WT and *pdcd11* mutants.

G-H. The numbers of terminal deoxynucleotidyl transferase dUTP nick end labeling and Pu.1:GFP double positive cells (n = 20 each).

I-J. WISH (I) and qPCR (J) assay of zebrafish *tgfb1b* expressed in 22hpf WT and *pdcd11* mutants.

The number positioned in the lower right corner of Figure S2A to S2D represent the number of zebrafish embryos shown positive phenotypes versus the total number of embryos examined. Scale bar: 100μm. n.s, not significant (Student’s t-test).

**Figure S3. Hyperactivated inflammatory response in *pdcd11* mutants is restored with NF-κB inhibition.**

A. Rescue effects on expression of *p53* pathway genes with Ribosome domain (Rib), or Coil overexpression.

B. Rescue effects on *mfap4* expressing macrophages with Ribosome domain (Rib) overexpression.

C. Expression pattern of *mfap4* in the 52hpf brain of WT and *pdcd11* mutants with Coil mRNA overexpression, or coil overexpressed in macrophages (*mpeg1* promoter) or neurons (*Huc* promoter).

D. Representative image of three independent experiments showing P65^536^ levels in WT and *pdcd11* mutants with NF-κB inhibitors treatment.

E. Inflammatory genes expression in 52hpf WT and *pdcd11* mutants treated with NF-κB inhibitor.

F. Expression of inflammatory genes with Coil mRNA overexpression, *mpeg1* promoter driven Coil, and *Huc* promoter driven Coil expression in 52hpf WT and *pdcd11* mutants.

The number positioned in the lower left corner of Figure S3B and S3C represent the number of zebrafish embryos shown positive phenotypes versus the total number of embryos examined. Scale bar: 100μm. Means ± SEM are shown for three independent experiments. n.s, not significant, ^*^*P* < 0.05; ^**^*P* < 0.01; ^***^*P* < 0.001 (Student’s t-test).

**Figure S4. PDCD11 overexpression promotes NF-κB activation.**

A. The percentage of cells with NF-κB activation were calculated according to Coil nuclear expression or not.

B. Percentage of cells with nuclear expressed c-Rel were analyzed regarding Coil expression or not respectively.

C. Percentage of cells with nuclear expressed P105 were analyzed regarding Coil expression or not respectively.

D. Immunofluorescence assay of the Pdcd11 expression in mpo labeled transgenic line.

Scale bar: 50μm. Means ± SEM are shown for three independent experiments. ^*^*P* < 0.05; ^**^*P* < 0.01; ^***^*P* < 0.001 (Student’s t-test).
