# Supplementary figures and images for: Yolk sac-derived Pdcd11-positive cells modulate zebrafish microglia differentiation through the NF-κB-Tgfβ1 pathway
Source: Cell Death Differ. 2020 Jul 24;28(1):170–83. doi: 10.1038/s41418-020-0591-3 (PMC7853042; doi:10.1038/s41418-020-0591-3)

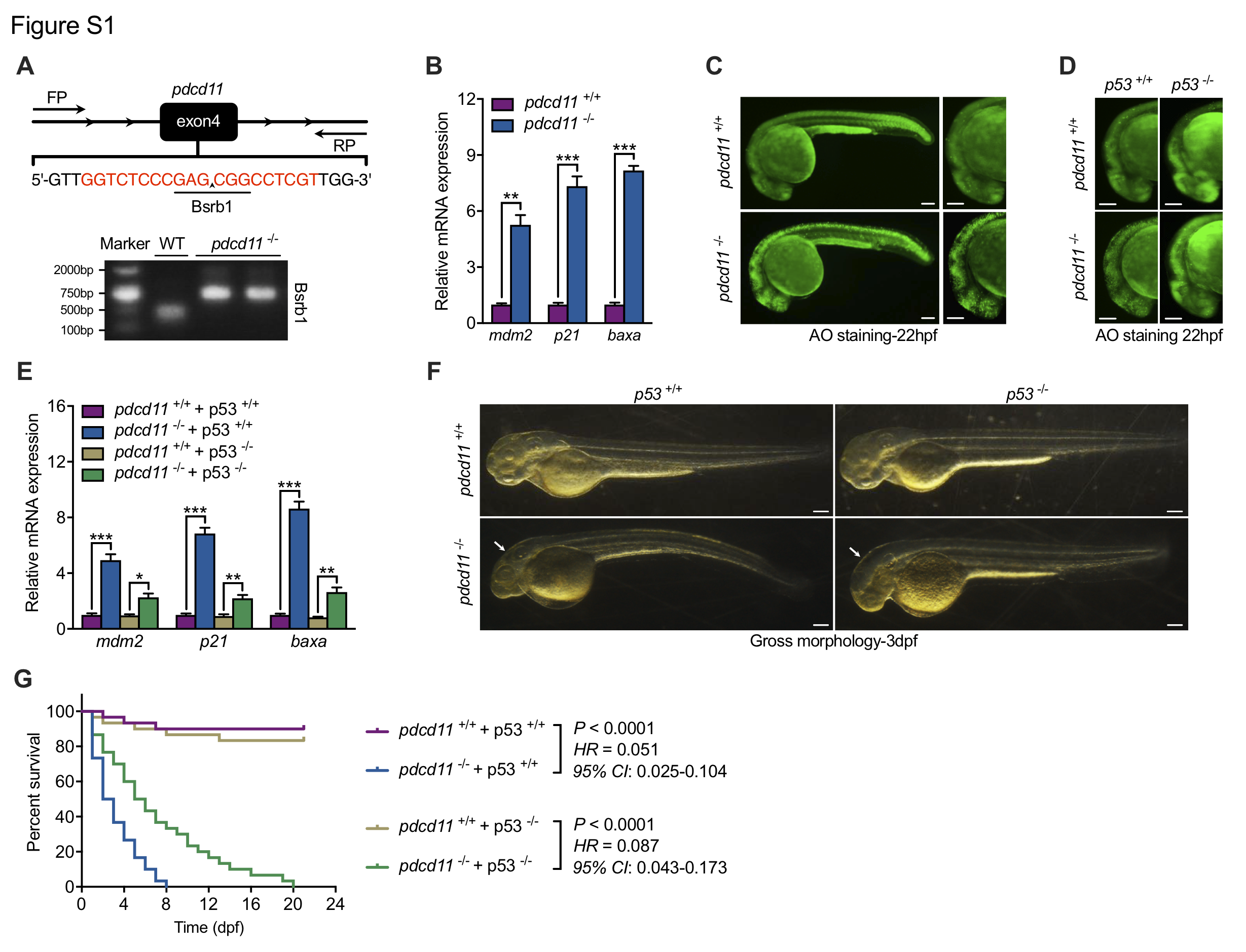

Supplement: Supplementary file 2 — Supplemental Figure 1 [file 41418_2020_591_MOESM2_ESM.png]

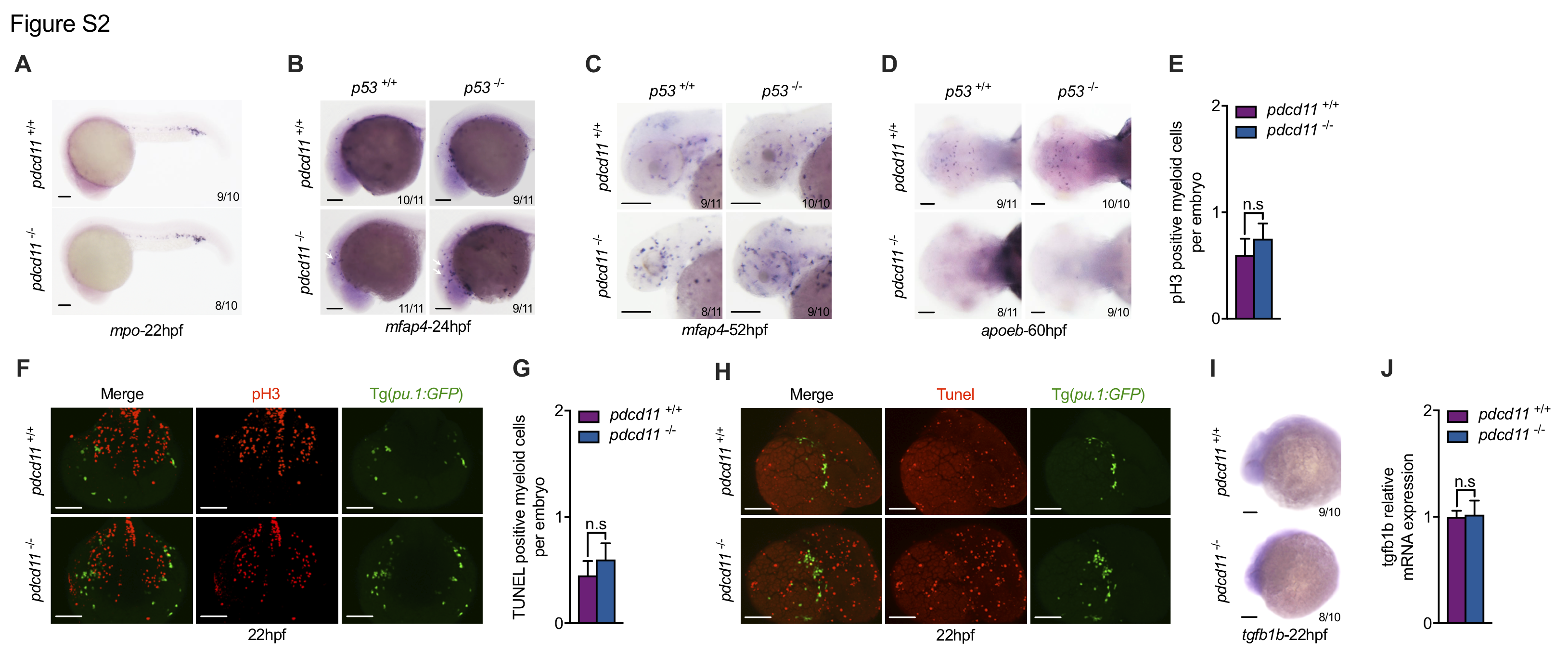

Supplement: Supplementary file 3 — Supplemental Figure 2 [file 41418_2020_591_MOESM3_ESM.png]

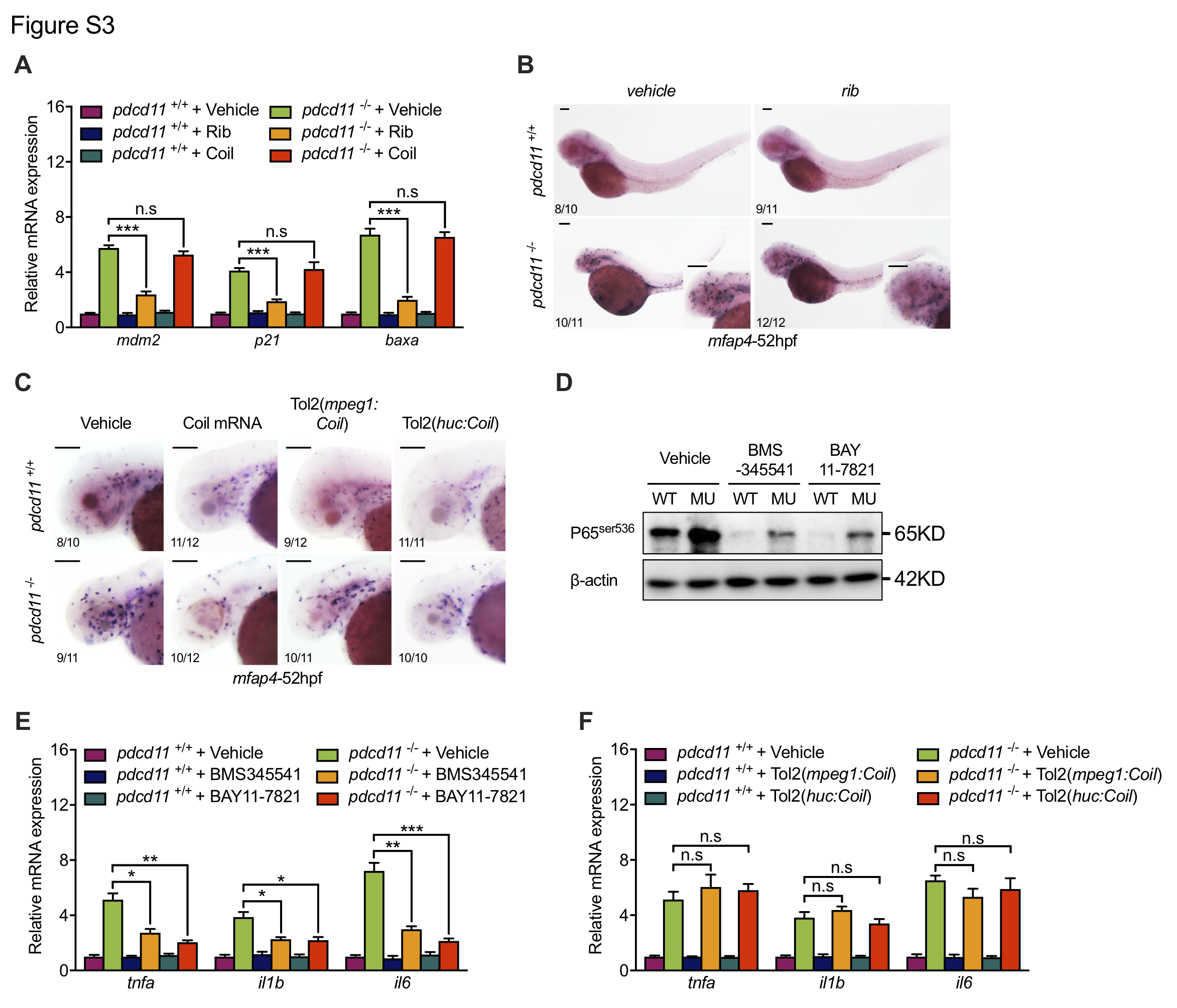

Supplement: Supplementary file 4 — Supplemental Figure 3 [file 41418_2020_591_MOESM4_ESM.png]

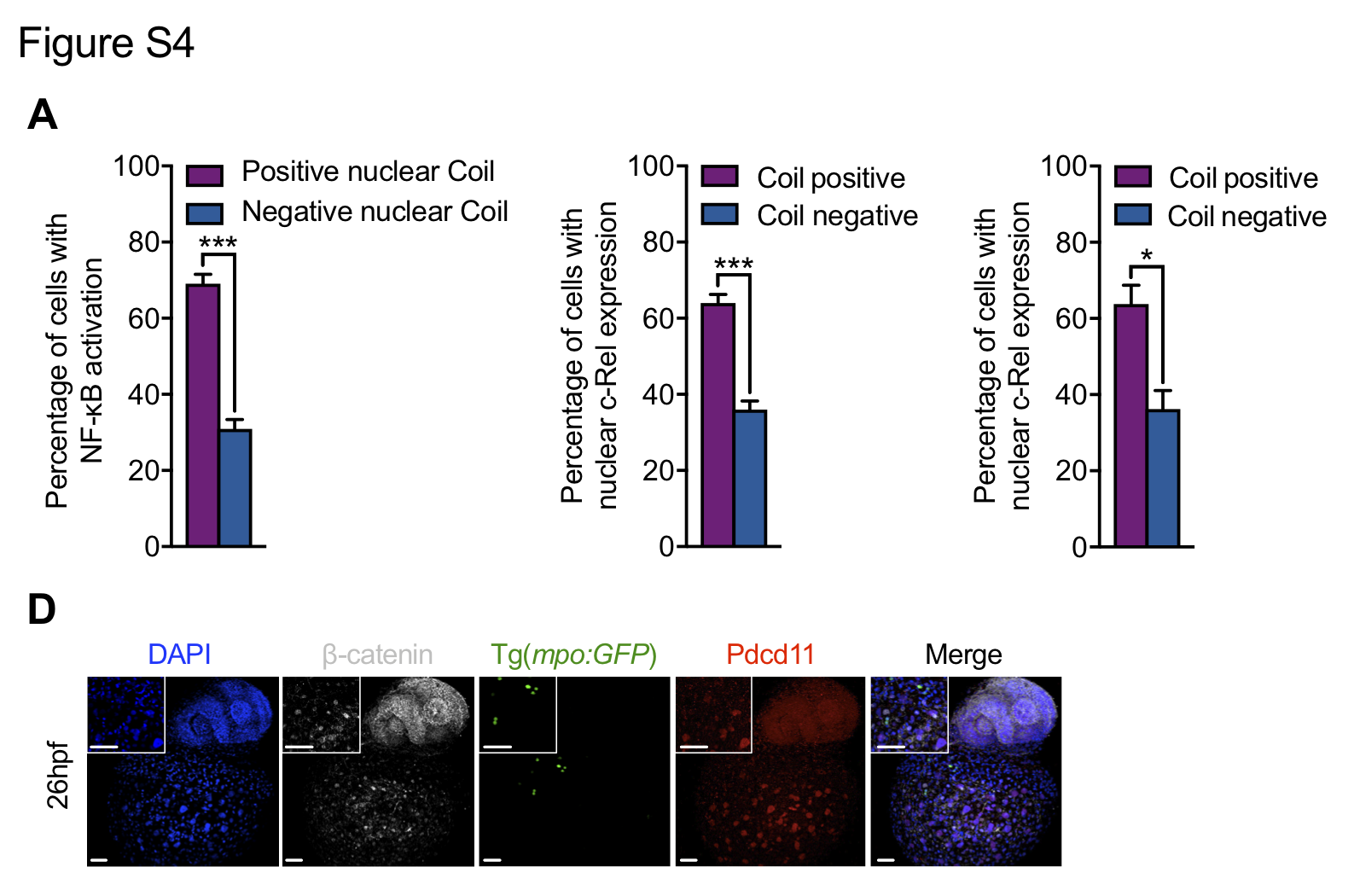

Supplement: Supplementary file 5 — Supplemental Figure 4 [file 41418_2020_591_MOESM5_ESM.png]
