# Supplementary material for: Yolk sac-derived Pdcd11-positive cells modulate zebrafish microglia differentiation through the NF-κB-Tgfβ1 pathway
Source: Cell Death Differ. 2020 Jul 24;28(1):170–83. doi: 10.1038/s41418-020-0591-3 (PMC7853042; doi:10.1038/s41418-020-0591-3)
Supplement: Supplementary file 7 — Supplemental Table 2 [file 41418_2020_591_MOESM7_ESM.docx]

**supplement table 1. probe and primer sequences in this study**

| **genes** | **primer sequences** | |
| --- | --- | --- |
| **probe for *in vitro* transcription** | | |
| apoeb-probe | forward | 5'-tggaccgtttctggcagtat-3' |
| apoe-probe-t7 | reverse | 5'-taatacgactcactatagggtggctcatgtatggctggaa-3' |
| zf-il6-probe | forward | 5'-gcactcctctcctcaaacct-3' |
| zf-il6-probe-t7 | reverse | 5'-taatacgactcactatagggttagttcttgtcagcgctgc-3' |
| zf-il1b-probe | forward | 5'-cgtcatccaagagcgtgaag-3' |
| zf-il1b-probe-t7 | reverse | 5'-taatacgactcactatagggaatcttcatacgcggtgctg-3' |
| zf-mfap4-probe | forward | 5'-atgcccttcgactgttctga-3' |
| zf-mfap4-probe-t7 | reverse | 5'-taatacgactcactatagggcctgctcctccatcagtgaa-3' |
| zf-pdcd11-probe | forward | 5'-cgctgctgtcaaaagacact-3' |
| zf-pdcd11-probe-t7 | reverse | 5'-taatacgactcactatagggcggcttgcttttgacttcct-3' |
| zf-csf1b-probe | forward | 5'- ctgtgtgttcccctctccat-3' |
| zf-csf1b-probe-t7 | reverse | 5'-taatacgactcactatagggaggtccagttaacaggggtg -3' |
| zf-ctsba-probe | forward | 5'-agctcttcaagaacggtcca-3' |
| zf-ctsba-probe-t7 | reverse | 5'-taatacgactcactatagggagtccaatgagcaggtccaa-3' |
| zf-tgfb1a-probe | forward | 5'-tggtgaggaagaagcggatt-3' |
| zf-tgfb1a-probe-t7 | reverse | 5'-taatacgactcactatagggtgaaccctgcagccattcta-3' |
| zf-cxcr3.1-probe | forward | 5'- aggagtggatctttggcaca -3' |
| zf-cxcr3.1-probe-t7 | reverse | 5'- taatacgactcactatagggagctgatgaagaaggccagt -3' |
| zf-cxcr3.2-probe | forward | 5'- aactggagctttgttctcgc -3' |
| zf-cxcr3.2-probe-t7 | reverse | 5'- taatacgactcactatagggctgcaagctgtcagtcatcc -3' |
| zf- tgfb1b-probe | forward | 5'- aactacgactgcccaaggaa -3' |
| zf- tgfb1b-probe-t7 | reverse | 5'- taatacgactcactatagggatcaaacgacacccacttgc -3' |
| **pdcd11 promoter** | | |
| dr-pdcd11-promoter-xho1 | forward | 5'-ggcctcgagcaataagttaac-3' |
| dr-pdcd11-promoter-ecor1 | reverse | 5'-atagaattctgtaaacaccga-3' |
| hu-tgfb1-promoter-kpn1 | forward | 5'-ttggtaccttgtttcccagcctgactct-3' |
| hu-tgfb1-promoter-xho1 | reverse | 5'-ttctcgagcgaggtctggggaaaagtct-3' |
| hu-coil-ecor1 | forward | 5'-atggtaccttgtttcccagcctgactct-3' |
| hu-coil-xho1 | reverse | 5'-ttctcgagctagtcctctagcactgagc-3' |
| **plasmid construction** |  |  |
| hu-flag-coil-ecor1 | forward | 5'-ttgaattccggaattcatggattacaaggatgacgacgataaggcttccttggaagggcaact-3' |
| hu-flag-ter-ecor1 | forward | 5'-ttgaattccggaattcatggattacaaggatgacgacgataaggcagccagtcaccgcgt-3' |
| hu-ter-xho1 | reverse | 5'-ttctcgagctagtcctctagcactgagc-3' |
| hu-coil coil-xho1 | reverse | 5'-ttctcgagctaagcctggctcctccgcagaag-3' |
| hu-tgfβ1-ecor1 | forward | 5'-ttgaattcatgccgccctccgggct-3' |
| hu-tgfβ1-xho1 | reverse | 5'-ttctcgaggctgcacttgcaggagc-3' |
| dr-tgfβ1-ecor1 | forward | 5'-ttgaattcatgaggttggtttgcttg-3' |
| dr-tgfβ1-xho1 | reverse | 5'-ttctcgagactgcacttgcagttcc-3' |
| hu-p105-ecor1 | forward | 5'-gggaattcatggcagaagatgatc-3' |
| hu-p105-xho1 | reverse | 5'-ttctcgagaattttgccttctag-3' |
| hu-c-rel-ecor1 |  | 5'-ttgaattcatggcctccggtgcgtata-3' |
| hu-c-rel-xho1 | forward | 5'-ttctcgagtacttgaaaaaattcatatggaaa-3' |
| hu-c-rel-rhd-xho1 | reverse | 5'-ttctcgagctggcacagtttctggaaaagc-3' |
| hu-c-rel-tad-ecor1 | reverse | 5'-ttgaattcatggatcacgttaattttcc-3' |
| zf-pdcd11-cas9-screen | forward | 5'-ttgacaggagggcgaataatt-3' |
| zf-pdcd11-cas9-screen | forward | 5'-tgtctcccttatatgacccattaac-3' |
| hu-p65-bamh1 | reverse | 5'-ttggatccatggacgaactgttccccc-3' |
| hu-p65-xho1 | forward | 5'-ttctcgagggagctgatctgactcagc-3' |
| hu-p65-rhd-xho1 | reverse | 5'-ttctcgaggctcttgaaggtctcatatg-3' |
| hu-p65-tad-bamh1 | reverse | 5'-ttggatccatgatcatgaagaagagtcctttc-3' |
| **qpcr** |  |  |
| zf-il6-qpcr | forward | 5'-cgtaaagagtctccttggcg-3' |
| zf-il6-qpcr | reverse | 5'-gccgtcatgttcaccatctc-3' |
| zf-il1b-qpcr | forward | 5'-gcacatcaaaccccaatcca-3' |
| zf-il1b-qpcr | reverse | 5'-gcactgaatccaccacgttc-3' |
| zf-cxcr3.1-qpcr | forward | 5'-acagacgtgaatctcaaagaca-3' |
| zf-cxcr3.1-qpcr | reverse | 5'-aagaccacagatgcctccaa-3' |
| zf-tnfa-qpcr | forward | 5'-tggaagtgtgctgagactca-3' |
| zf-tnfa-qpcr | reverse | 5'-ctggtcctggtcatctctcc-3' |
| zf-cxcr3.2-qpcr | forward | 5'-cctaacatctcgtgcccact-3' |
| zf-cxcr3.2-qpcr | reverse | 5'-aatctgtaggcgctgagacc-3' |
| zf-cst3-qpcr | forward | 5'-atggcccagtacaacagaca-3' |
| zf-cst3-qpcr | reverse | 5'-tcaactccaccctttctgca-3' |
| zf-f11r.1-qpcr | forward | 5'-gactgtgaggtgtctggaag-3' |
| zf-f11r.1-qpcr | reverse | 5'-gccggacattactgcttgtt-3' |
| zf-tlr5a-qpcr | forward | 5'-acacgatggattaatgggcg-3' |
| zf-tlr5a-qpcr | reverse | 5'-gcgaagtagccatcagttga-3' |
| zf-atf3-qpcr | forward | 5'-cagcagcaaaatgtcggaac-3' |
| zf-atf3-qpcr | reverse | 5'-ttggttcttcagctcctcga-3' |
| zf-ctsl.1-qpcr | forward | 5'-cagtgctgccagtctttctc-3' |
| zf-ctsl.1-qpcr | reverse | 5'-ctgacggtgtgactcttcct-3' |
| zf-csf1a-qpcr | forward | 5'-tcacagagatgcatcccttca-3' |
| zf-csf1a-qpcr | reverse | 5'-tgcgaatcacttcctcagct-3' |
| zf-csf1b-qpcr | forward | 5'-gtccacttcagatacgtgcg-3' |
| zf-csf1b-qpcr | reverse | 5'-cctcctccagttcctcgttt-3' |
| zf-plxdc2-qpcr | forward | 5'-agaacactggctgatgacga-3' |
| zf-plxdc2-qpcr | reverse | 5'-tctgctcatagggtcactgg-3' |
| zf-slc1a3a-qpcr | forward | 5'-ccagaacatctccagcaacg-3' |
| zf-slc1a3a-qpcr | reverse | 5'-cttcacctcccgatacgaca-3' |
| zf-sall3a-qpcr | forward | 5'-cagctgaagaatgtcccgtc-3' |
| zf-sall3a-qpcr | reverse | 5'-gctctcatttccgctgtctg-3' |
| zf-rasgrp3-qpcr | forward | 5'-aagagtttcgagatgcagcc-3' |
| zf-rasgrp3-qpcr | reverse | 5'-cctgcttctttcgctgagtc-3' |
| zf-tgfbr1a-qpcr | forward | 5'-ctgctgcaacactcacatgt-3' |
| zf-tgfbr1a-qpcr | reverse | 5'-cacaaagctcagcacacaga-3' |
| zf-lrrc3b-qpcr | forward | 5'-tgagtgctctggatgctgaa-3' |
| zf-lrrc3b-qpcr | reverse | 5'-tgacagccggccaacata-3' |
| zf-adamts1-qpcr | forward | 5'-gaccgtcacccagaacacta-3' |
| zf-adamts1-qpcr | reverse | 5'-gtgccaacatctgccatcc-3' |
| zf-olfml3b-qpcr | forward | 5'-gagcagcaggtgacgtttg-3' |
| zf-olfml3b-qpcr | reverse | 5'-ccttggtccacatgccctta-3' |
| zf-tmem119b-qpcr | forward | 5'-ccagatcatcttcagtcggc-3' |
| zf-tmem119b-qpcr | reverse | 5'-gactggggtggcattagagt-3' |
| zf-tgfbr1b-qpcr | forward | 5'-cccgacatcaaagcattccc-3' |
| zf-tgfbr1b-qpcr | reverse | 5'-tggcagatgtagaagacggt-3' |
| zf-sall1a-qpcr | forward | 5'-acattgtcactgcaactgttaca-3' |
| zf-sall1a-qpcr | reverse | 5'-gagtcggactggaaatgctg-3' |
| zf-mdm2-qpcr | forward | 5'-caggaggaggagaagcagtg-3' |
| zf-mdm2-qpcr | reverse | 5'-caatcacgcaccaagacagg-3' |
| zf-p21-qpcr | forward | 5'-aagcgcaaacagaccaacat-3' |
| zf-p21-qpcr | reverse | 5'-tgtgtgtgtgtgtgtctcct-3' |
| zf-baxa-qpcr | forward | 5'-atcagggaacagggtggatg-3' |
| zf-baxa-qpcr | reverse | 5'-ttgtcatcctcgggttcaca-3' |
